# Fat in the lumbar multifidus muscles - predictive value and change following disc prosthesis surgery and multidisciplinary rehabilitation in patients with chronic low back pain and degenerative disc: 2-year follow-up of a randomized trial

**DOI:** 10.1186/s12891-017-1505-5

**Published:** 2017-04-04

**Authors:** Kjersti Storheim, Linda Berg, Christian Hellum, Øivind Gjertsen, Gesche Neckelmann, Ansgar Espeland, Anne Keller, on behalf of the Norwegian Spine Study Group, Odd-Inge Solem, Jens Munch-Ellingsen, Franz Hintringer, Anita Dimmen Johansen, Guro Kjos, Øystein P. Nygaard, Lars Gunnar Johnsen, Ivar Rossvoll, Hege Andresen, Helge Rønningen, Kjell Arne Kvistad, Magne Rø, Bjørn Skogstad, Janne Birgitte Børke, Erik Nordtvedt, Gunnar Leivseth, Sjur Braaten, Turid Rognsvåg, Gunn Odil Hirth Moberg, Jan Sture Skouen, Lars Geir Larsen, Vibeche Iversen, Ellen H. Haldorsen, Elin Karin Johnsen, Kristin Hannestad, Endre Refsdal, Oliver Grundnes, Jens Ivar Brox, Vegard Slettemoen, Kenneth Nilsen, Kjersti Sunde, Helenè E. Skaara, Berit Johannessen, Anna Maria Eriksdotter

**Affiliations:** 1 Research and Communication unit for musculoskeletal disorders (FORMI), Oslo University Hospital Ullevål, Postbox 4956, Nydalen, 0424 Oslo Norway; 2 Faculty of Medicine, University of Oslo, Postbox 1078, Blindern, 0316 Oslo Norway; 3 Department of Radiology, Haukeland University Hospital, Postbox 1400, 5021 Bergen, Norway; 4 Section for Radiology, Department of Clinical Medicine, University of Bergen, Postbox 7804, N-5020 Bergen, Norway; 5 Department of Radiology, Nordland Hospital, Postbox 1480, 8092 Bodø, Norway; 6 Institute of Clinical Medicine, UiT, The Arctic University of Norway, Postbox 6050, Langnes, 9037 Tromsø Norway; 7 Department of Orthopaedics, Oslo University Hospital Ullevål, Postbox 4956, Nydalen, 0424 Oslo Norway; 8 Department of Radiology and Nuclearmedicine, Oslo University Hospital Rikshospitalet, Postbox 4950, Nydalen, 0424 Oslo Norway; 9 Department of Physical Medicine and Rehabilitation, Oslo University Hospital Ullevål, Postbox 4956, Nydalen, 0424 Oslo Norway; 10 Center for Rheumatology and Spine Diseases, National Hospital, 2600 Glostrup, Denmark

**Keywords:** Multifidus muscle fat, Predictive value, Change over time, Chronic degenerative low back pain, Multidisciplinary rehabilitation, Physiotherapy, Surgery, Total disc replacement

## Abstract

**Background:**

Evidence is lacking on whether fat infiltration in the multifidus muscles affects outcomes after total disc replacement (TDR) surgery and if it develops after surgery. The aims of this study were 1) to investigate whether pre-treatment multifidus muscle fat infiltration predicts outcome 2 years after treatment with TDR surgery or multidisciplinary rehabilitation, and 2) to compare changes in multifidus muscle fat infiltration from pre-treatment to 2-year follow-up between the two treatment groups.

**Methods:**

The study is secondary analysis of data from a trial with 2-year follow-up of patients with chronic low back pain (LBP) and degenerative disc randomized to TDR surgery or multidisciplinary rehabilitation. We analyzed (aim 1) patients with both magnetic resonance imaging (MRI) at pre-treatment and valid data on outcome measures at 2-year follow-up (predictor analysis), and (aim 2) patients with MRI at both pre-treatment and 2-year follow-up. Outcome measures were visual analogue scale (VAS) for LBP, Oswestry Disability Index (ODI), work status and muscle fat infiltration on MRI. Patients with pre-treatment MRI and 2-year outcome data on VAS for LBP (*n* = 144), ODI (*n* = 147), and work status (*n* = 137) were analyzed for prediction purposes. At 2-year follow-up, 126 patients had another MRI scan, and change in muscle fat infiltration was compared between the two treatment groups. Three radiologists visually quantified multifidus muscle fat in the three lower lumbar levels on MRI as <20% (grade 0), 20–50% (grade 1), or >50% (grade 2) of the muscle cross-section containing fat. Regression analysis and a mid-P exact test were carried out.

**Results:**

Grade 0 pre-treatment multifidus muscle fat predicted better clinical results at 2-year follow-up after TDR surgery (all outcomes) but not after rehabilitation. At 2-year follow-up, increased fat infiltration was more common in the surgery group (intention-to-treat *p* = 0.03, per protocol *p* = 0.08) where it was related to worse pain and ODI.

**Conclusions:**

Patients with less fat infiltration of multifidus muscles before TDR surgery had better outcomes at 2-year follow-up, but findings also indicated a negative influence of TDR surgery on back muscle morphology in some patients. The rehabilitation group maintained their muscular morphology and were unaffected by pre-treatment multifidus muscle fat.

**Trial registration:**

NCT 00394732 (retrospectively registered October 31, 2006).

## Background

During the past 25 years, total disc replacement (TDR) surgery has become an option for selected patients with chronic low back pain (LBP) traditionally treated conservatively or with spinal fusion [1]. Randomized trials have found clinical outcome of TDR to be at least equivalent to that of fusion [2]. In the first study to compare TDR to non-surgical treatment, TDR was more effective than multidisciplinary rehabilitation at 2-year follow-up, based on patient reported outcomes like disability, pain, quality of life, and patient satisfaction [3].

A variety of muscles, including the superficial and deep layers of the paraspinal muscles, contributes to stabilization and movement of the spine [4–8]. Altered paraspinal muscle morphology – such as fat infiltration in the lumbar multifidus muscles [9] – may be related to back pain [9–15] and low physical activity [15]. Physical exercises can improve and maintain muscular fitness [16] and resistance exercise can prevent fat infiltration in skeletal muscle [17]. However, it is not clear whether such muscle alterations affect outcomes after TDR surgery. In the only previous study of this issue, less paraspinal muscle fat preoperatively was related to better results 2 years after surgery [18]. It is also unclear whether TDR surgery affects the paraspinal muscles. Surgical techniques more invasive to the back muscles (like posterior lumbar fusion) can change back muscle morphology, possibly explained by muscle denervation [19–25]. TDR surgery with anterior access is hypothesized to minimize back muscle injury and thereby prevent nerve injury and subsequent altered muscle morphology. Another possible advantage of TDR surgery is maintained mobility at the operated level, which also may be favorable for the back muscles [26].

New surgical interventions should be compared with conservative treatment [27] and the present study is an analysis of the lumbar multifidus muscles of patients included in the first randomized trial of TDR surgery with such a design [3]. Our *a priori* aims were 1) to investigate whether pre-treatment multifidus muscle fat infiltration predicts outcome 2 years after treatment with TDR surgery or multidisciplinary rehabilitation, and 2) to compare changes in fat infiltration between the two treatment groups from pre-treatment to 2-year follow-up.

## Methods

This is a secondary analysis of patients included in a randomized trial evaluating the effect of surgery with disc prosthesis versus rehabilitation [3]. The trial included 173 patients who were randomized and treated with TDR surgery or multidisciplinary rehabilitation between May 2004 and September 2007: 86 were randomized to surgery and 87 to rehabilitation. Patients underwent pre-treatment magnetic resonance imaging (MRI) of the lumbar spine 0–12 months prior to inclusion and a follow-up MRI with clinical investigation 2 years after treatment. The Regional Committees for Medical Research Ethics in east Norway approved the study (43-04013) and all participants gave written informed consent. The trial was conducted in accordance with the Helsinki Declaration and the ICH-GCP guidelines and registered at www.clinicaltrial.gov under the identifier NCT 00394732.

### Eligibility criteria and study sample

As detailed elsewhere [3], inclusion criteria for the main trial were age 25–55 years, LBP as the main symptom for at least 1 year, structured physiotherapy or chiropractic treatment for at least 6 months without sufficient effect, Oswestry Disability Index (ODI) ≥30%, and degenerative disc at L4/L5 and/or L5/S1 defined by the following MRI findings: A) ≥40% reduction of disc height [28] and/or B) at least two of these three findings: Modic changes type I and/or II [29], posterior high intensity zone (HIZ) in the disc [30], and dark/black nucleus pulposus on T2-weighted images (i.e. grade 2 or 3 signal intensity changes) [31]. Exclusion criteria were any of the four MRI findings in A) or B) at any higher lumbar level (L1-L4), spondylolysis, spondylolisthesis, arthritis (e.g., ankylosing spondylitis), osteoporosis, prior fracture L1-S1, prior spinal fusion, deformity, osteoporosis, symptomatic disc herniation/spinal stenosis, generalized chronic pain, ongoing psychiatric or somatic disease that excluded either one or both treatment alternatives, drug abuse, or inability to understand Norwegian.

**Fig. 1:**
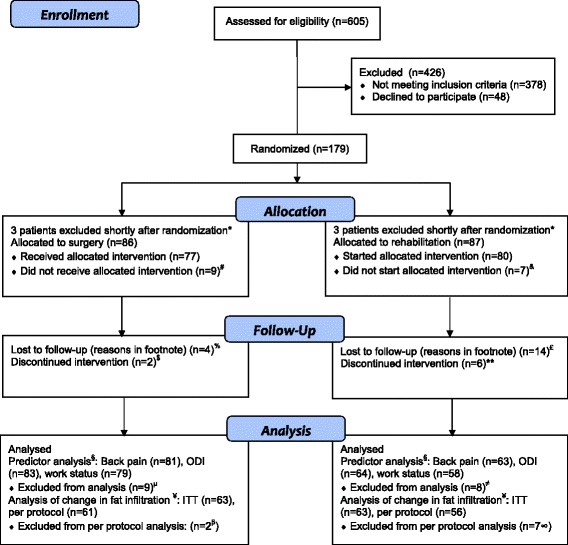
CONSORT flow diagram. * Heart attack some days after randomization (*n* = 1), obvious exclusion criterion discovered some days after randomization (earlier large abdominal operation (*n* = 1)), degenerative change insufficient to satisfy inclusion criteria (*n* = 2) or present in more than two lower lumbar discs (*n* = 2)). # Changed their mind and declined surgery after randomization (3 had social reasons for not receiving treatment, 1 had work related economic reasons, and 5 wanted guaranteed success). & Changed their mind after randomization and did not attend the rehabilitation program (2 had work-related economic reasons, 1 was treated elsewhere with surgery for lumbar disc herniation, 1 had social reasons, and 2 needed to travel long distances/could not stay away from home). % Dropped out after total disc replacement (TDR) surgery (1 had serious complications with a vascular injury and leg amputation, 2 did not want to attend the follow-up and 1 could not be contacted after surgery). £ 6 patients dropped out during the rehabilitation program (1 did not find the program good enough, 1 had lumbar disc herniation during treatment and underwent microdiscectomi, 1 did not manage to go through the training program, 1 developed diabetes during or just before treatment, 1 had psychosocial reasons, and 1 had hypertension and the family doctor did not recommend training), 8 dropped out after completing the treatment (1 took part in another study, 1 patient did not complete the questionnaire, 1 patient moved, 1 patient died of cancer, 3 did not want to attend the follow-up, and there was 1 for whom the reason was unknown). $ Two patients underwent surgery with instrumented fusion before 2-year follow-up. ** One patient crossed over to surgery between 6 months and 1 year and five patients between 1 year and 2 years. Five patients underwent TDR surgery and one patient fusion. § Subjects relevant for analysis were patients with both a pre-treatment MRI and valid score for back pain, Oswestry Disability Index (ODI) score and data on work status at 2-year follow-up. Patients randomized to rehabilitation who crossed over and underwent TDR surgery before 2-year follow-up within (*n* = 5) or outside (*n* = 5) the study setting are analyzed in the surgery group, patients who refused TDR surgery and underwent rehabilitation were analyzed in the rehabilitation group (*n* = 2), according to as-treated principles. μ Refused surgery (*n* = 7), re-operated upon with a fusion (*n* = 2). ≠ Did not start the rehabilitation program (*n* = 7), received a primary fusion (*n* = 1). ¥ Randomized design (RCT) includes patients with MRI at both pre-treatment and 2-year follow-up. β Re-operated upon with a fusion. ∞ Crossed over to surgery (*n* = 5 to TDR and *n* = 1 to fusion), did not complete the rehabilitation program (*n* = 1)

In the present study, we analyzed: 1) patients with both a pre-treatment MRI and valid visual analogue scale (VAS) score for LBP, ODI score and data of work status at 2-year follow-up (predictor analysis), and: 2) patients with MRI at both pre-treatment and 2-year follow-up (to compare change in fat infiltration over time between treatment groups). See CONSORT flow diagram for details of patients included in these secondary analyses (Fig. 1). In the predictor analysis, patients crossing over from rehabilitation to TDR surgery during the 2-year follow-up period were analyzed in the surgical group, and patients randomized to surgery who refused surgery and underwent rehabilitation were analyzed in the rehabilitation group according to as-treated principles. Patients not undergoing allocated interventions and patients operated upon with a fusion were excluded from the predictor analysis. When comparing change in fat infiltration over time between treatment groups, all available patients were examined according to the intention-to-treat (ITT) analysis, but in a secondary per protocol analysis we excluded patients deviating from the study protocol (Fig. 1).

### Study interventions

Interventions have been described in detail elsewhere [3]. *The rehabilitation intervention* was based on the treatment model described by Brox et al and consisted of supervised physical exercise with a cognitive approach [32]. Patients were treated in groups by a multidisciplinary team of physiotherapists and specialists in physical medicine and rehabilitation (plus other professions if required) at the hospitals’ outpatient clinics for about 60 h over 3–5 weeks / 12–15 days. The multidisciplinary rehabilitation program included general exercise for increasing overall fitness (cardiovascular, strength (particularly thighs, back- and abdominal muscles), flexibility, coordination, body awareness and relaxation), and for specific individual needs (strength (including the transverse abdominal muscles and multifidus muscles, flexibility, endurance, etc.). Examples of general exercise are group exercise accompanied by music (“Aerobics”), circuit training, swimming / water games, biking, Nordic walking, treadmill walking, cross country skiing and games (i.e. ball games). Patients had two or three workout sessions per treatment day, at least one “heavy” and one “light” and one group based and one individual session. Intensity was gradually increased during the rehabilitation period. Physiotherapists supervised most exercise, but patients were also encouraged to exercise by themselves at home and after ended rehabilitation period. Overall goal for the training was to increase patients’ belief and confidence in being able to perform daily activities of life and to increase functional capacity although the back may hurt. *The surgical intervention* was replacement of the degenerative intervertebral lumbar disc with an artificial lumbar disc (ProDisc II, Synthes Spine). There were no major postoperative restrictions and patients were not referred for postoperative physiotherapy, but at 6-week follow-up they could be referred for physiotherapy if required (emphasizing general mobilization and exercise). All patients were treated within 3 months after randomization.

### Measurement of outcomes and possible predictors

The only variable tested for predictive value (independent variable) was multifidus muscle fat on MRI. MRI performed at the different trial sites typically included sagittal T1- and T2-weighted images and axial images of the three lower lumbar levels (T2-, T1-, and/or proton density-weighted); image characteristics are given in Table 1. Typically, slice thickness was 3 − 5 mm, interslice gap 0 − 1.4 mm, field of view 28 − 35 cm for sagittal and 17 − 30 cm for axial images, and matrix 512 × 512 (varied from 160 × 256 to 1024x1024). The images were obtained directly in Digital Imaging and Communications in Medicine (DICOM) format or, for seven examinations, as digitized printed film hard copies stored in DICOM format.

**Table 1:** Magnetic resonance imaging characteristics

Characteristics	Predictor analysis (137 patients, 137 examinations)	Analysis of change in fat infiltration (126 patients, 252 examinations)
1.5 T	121 / 137 examinations (88%)	235 / 252 examinations (93%)
Sagittal T1-weighted images	128 / 137: FSE (TR / TE, 350 − 911 ms / 7.4 − 20 ms) 8 / 137: FLAIR images (TR / TE, 1984 − 2130 ms / 20 − 22.1 ms)	244 / 252 FSE (TR / TE, 360 − 911 ms / 7 − 22 ms) 7 / 252 FLAIR images (TR / TE, 1984 − 2130 ms / 20 − 22 ms)
Sagittal T2-weighted images	136 / 137 FSE (TR / TE, 2511 − 4760 ms / 70 − 140 ms)	251^a^ / 252 FSE (TR / TE, 2000 − 5070 ms / 70 − 140 ms) and/or DRIVE images (FSE with 90° Flip-Back Pulse: TR / TE 700 ms / 135 − 140 ms): 236 FSE only (126 pre-treatment and 110 2-year) 12 DRIVE only (all 2-year), and 3 both FSE and DRIVE (all 2-year)
Axial images at L3/L4, L4/L5 and L5/S1	134 / 137 (105 T2-weighted, 27 T1-weighted, and 19 proton density-weighted images)	247 / 252 (213 T2-weighted, 31 T1-weighted, and 19 proton density-weighted images)

*TR* repetition time, *TE* echo time, *FLAIR* fluid-attenuated inversion-recovery, *FSE* fast spin echo

^a^one examination lacked sagittal T2-weighted FSE images at 2 years but included sagittal STIR (short tau inversion-recovery) images

**Fig. 2:**
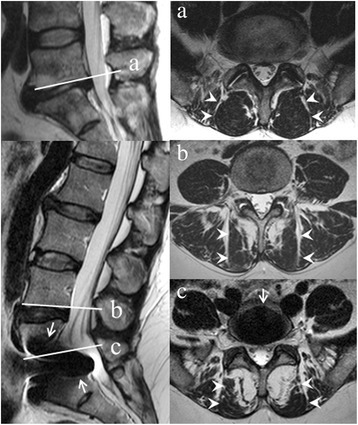
Grading of fat in the multifidus muscles on magnetic resonance imaging. Multifidus muscles (right, *arrowheads*) on axial T2-weighted images located as marked on sagittal T2-weighted images (left, *lines*) contain fat grade 0 at L5/S1 in one patient (**a**) and grade 1 at L4/L5 (**b**) and grade 2 at L5/S1 (**c**) in a different patient, whose disc prosthesis causes artefacts (*arrows*) that do not affect the grading. Grade 0: 0 or < 20% of total muscle cross-section (left plus right side) contains fat; grade 1: 20–50% of cross-section contains fat; grade 2: >50% of cross-section contains fat

Fat in the multifidus muscles was visually graded at levels L3/L4, L4/L5 and L5/S1 using the axial T2-weighted image (T1 if T2 was lacking) closest to an axial plane through the mid-sagittal posterior and anterior caudal corners of the upper vertebrae. The grading was based on the criteria used by Kjaer et al [9], but adjusted as recommended by Solgaard-Sørensen et al [33]: 0 = 0 or < 20% of total cross-section (left plus right side) contains fat, 1 = 20–50% of cross-section contains fat, 2= >50% of cross-section contains fat (Fig. 2). One radiologist, experienced in musculoskeletal MRI (reader A), and two neuroradiologists (readers B and C) from three different institutions evaluated the images independently, retrospectively, and blinded to clinical data. Each reader had more than 10 years’ experience in MRI of the lumbar spine. Readers A and B evaluated the images using the eFilm Lite software version 2.1.2 (Merge Healthcare, Hartland, Wisconsin), while reader C used the Agfa Impax 4.5 (Agfa HealthCare, Mortsel, Belgium). The images were anonymized and presented in random order.

Readers B and C independently graded pre-treatment fat in the lumbar multifidus muscles [9, 33] (kappa 0.42–0.51 for interobserver agreement on grade 0 versus grade 1 or 2 at L3/L4, L4/L5, and L5/S1 in the original sample of 170 MRIs). When a grading was agreed upon it was considered to be conclusive; otherwise the majority or median grading by readers A, B, and C determined the conclusive grading. The conclusive grade was 0 at all levels in 45.3% of the patients and 2 at one level in one patient; hence, patients were dichotomized as having grade 0 muscle fat at all evaluated levels versus grade 1 or 2 muscle fat at any level.

*Change in fat* in the multifidus muscles was rated by comparing the 2-year follow-up and the pre-treatment images. Any progress or regress of at least one grading category was reported. Readers A and B independently evaluated the images (the prevalence- and bias-adjusted kappa, used due to low prevalence of change, was 0.57 – 0.97 and indicated moderate to very good interobserver agreement on progress or not at L3/L4, L4/L5, and L5/S1 in the 126 patients studied). When reader A and B disagreed, reader C independently rated the actual level(s), and the majority or median rating was used.

*Outcome measures* (dependent variables) in the predictor analysis were pain, back specific function and work status at 2-year follow-up. Pain (LBP during the preceding week) was measured by a horizontal VAS, ranging from 0 to 100 mm with respective end anchors “no pain” and “worst pain imaginable” [34]. Back specific function was evaluated by the Norwegian ODI version 2.0 [35, 36]. The ODI ranges from 0 to 100, with a lower score indicating less severe disability. Work status at 2-year follow-up was obtained from the patients and from the National Insurance of employees and categorized into working/not working (working part or full time, being a student or homemaker = working).

*Possible effect moderators:* to test if the predictive value of fat in the multifidus muscles is influenced by effect moderators, the following other variables were controlled for (based on literature search): age, gender, leisure time physical activity [37], body mass index (BMI), and smoking. These data were collected at baseline.

### Statistical analysis

All data were analyzed using SPSS (version 18, SPSS Inc., Chicago, IL, USA). Dependent and independent variables and possible effect moderators were selected *a priori* before statistical analysis commenced. Patients were analyzed according to as-treated-principles in the predictor analysis and according to randomization (ITT) when comparing change in fat infiltration over time between treatment groups. A Chi-Square Test (Continuity Correction) was used to compare groups at baseline (proportion of patients with grade 0 *versus* grade 1 or 2 pre-treatment fat in the lumbar multifidus muscles) and to compare work status at baseline and at 2-year follow-up between patients with grade 0 *versus* grade 1 or 2 muscle fat in each treatment group. An independent-samples t-test (two-tailed) was used to compare pain and ODI at baseline and at 2 year follow-up between patients with grade 0 *versus* grade 1 or 2 fat in each treatment group.

Multiple regression analysis (linear) was carried out with pain and ODI as dependent variables, and logistic regression analysis was conducted with work status as dependent variable. The models were adjusted for age (years), gender, BMI, current smoking (yes/no), and leisure time physical activity [37] (grade 0–3), and assessed for normality, homoscedasticity, and collinearity by residuals and variance inflation factor (VIF). In addition, we adjusted for baseline pain in analysis of pain at 2 years as a dependent variable, and baseline ODI in analysis of ODI at 2 years as a dependent variable.

The Mid-P exact test was used to compare change in fat infiltration over time between treatment groups [38]. Changes were collapsed into reduced fat infiltration or no change *versus* increased fat infiltration (≥1 grade at 1 or more levels). A per protocol analysis excluding patients deviating from the study protocol was also conducted.

All *P* values are 2-sided and the significance level was 5%. No formal power calculation was conducted since the present study is a secondary analysis of patients included in a randomized controlled trial and therefore has a fixed sample size.

## Results

Out of 173 patients included in the original trial (of 605 patients screened for eligibility) [3], in these secondary predictor analyzes 144, 147, and 137 patients had pre-treatment MRI and valid 2-year data on back pain, ODI, and work status, respectively (Fig. 1). For comparing change in fat infiltration over time between treatment groups, 126 patients had MRI at both pre-treatment and 2-year follow-up and were included in the ITT analysis, and 117 were included in the per protocol analysis. Patients included in the predictor analyzes and in the between groups analysis of change in fat infiltration over time were similar at baseline (Table 2); mean age about 45 years with chronic LBP for well over 6 years, BMI just above the limit for normal weight, and ODI-score of about 42 points on average. Only 20% were gainfully employed.

**Table 2:** Patient characteristics at baseline

	Predictor analysis (*n* = 147)	Analysis of change in fat infiltration (*n* = 126)
Age (mean (SD))	41.0 (7.2)	41.6 (7.1)
Gender (women (n %))	77 (52.4)	65 (51.6)
BMI (mean (SD))	25.3 (3.2)	25.4 (3.2)
Current smoker (n % yes)	66 (44.9)	58 (46.0)
Previous back surgery (n % yes)^a^	44 (29.9)	37 (29.4)
Work status ^b^ (n % working)	31 (21.1)	25 (19.8)
Duration of back pain, years (mean (SD))	6.3 (5.9)	6.5 (6.1)
Daily consumption of opioids (n % yes)	34 (23.1)	30 (23.8)
ODI score, 0-100^c^ (mean (SD))	42.3 (9.0)	41.8 (8.4)
EQ-5D index, -0.59–1^d^ (mean (SD))	0.28 (0.30)	0.28 (0.30)
HSCL-25, 1-4^c^ (mean (SD))	1.80 (0.51)	1.81 (0.50)
FABQ-physical, 0-24^c^ (mean (SD))	13.2 (5.6)	13.3 (5.4)
FABQ-work, 0-42^c^ (mean (SD))	26.5 (10.6)	26.0 (10.4)
Back Pain, 0-100^c^ (mean (SD))	70.0 (14.9)	69.4 (15.0)
Leg Pain, 0-100^c^ (mean (SD))	44.5 (26.8)	47.0 (25.7)

*BMI* body mass index (weight in kilograms divided by height in meters squared), *ODI* Oswestry Disability Index, EQ-5D = EuroQol-5 Dimensions, *HSCL-25* Hopkins Symptom Checklist, *FABQ* Fear Avoidance Beliefs Questionnaire

^a^There were no differences in fat infiltration between patients with/without previous back surgery

^b^Working versus not working; including part-time work as working

^c^Lower scores indicate less severe symptoms

^d^Higher scores indicate better quality of life

Results for grading of pre-treatment fat in the multifidus muscles are shown in Tables 3 and 4. Almost half of the patients, 67 (45.6%) included in the predictor analysis and 59 (46.8%) of patients analyzed for change in fat infiltration over time, had fat grade 0 in the multifidus muscles at any evaluated level, about 5% had ≥20% fat in all three levels (Table 4). In only one patient at one level did >50% of the muscle cross-section (left plus right side) contain fat (Table 3). Fat was more common at the lower levels. Patients analyzed in the rehabilitation group and in the surgical group did not differ in presence of (yes/no), or number of levels of, pre-treatment multifidus muscle fat (valid for both predictor analysis and between groups analysis of change in fat over time).

**Table 3:** Visual grading of fat in the multifidus muscles in the two analysis- / treatment groups by level at pre-treatment

	Predictor analysis (*n* = 147)						Analysis of change in fat infiltration (*n* = 126)					
	Rehab (*n* = 64)			Surgery (*n* = 83)			Rehab (*n* = 63)			Surgery (*n* = 63)		
Grade^a^	0	1	2	0	1	2	0	1	2	0	1	2
L3/L4 (n / %)	60 (93.8)	4 (6.3)	0 (0)	77 (92.8)	6 (7.2)	0 (0)	60 (95.2)	3 (4.8)	0 (0)	58 (92.1)	5 (7.9)	0 (0)
L4/L5 (n / %)	48 (75.0)	16 (25.0)	0 (0)	61 (73.5)	22 (26.5)	0 (0)	47 (74.6)	16 (25.4)	0 (0)	49 (77.8)	14 (22.2)	0 (0)
L5/S1 (n / %)	33 (51.6)	31 (48.4)	0 (0)	36 (43.4)	46 (55.4)	1 (1.2)	32 (50.8)	31 (49.2)	0 (0)	29 (46.0)	33 (52.4)	1 (1.6)

^a^Grading according to the criteria Kjaer et al [9] and Solgaard et al [31]: Grade 0: 0 or <20% of total cross-section (left plus right side) contains fat, Grade 1: 20%–50% of cross-section (left plus right side) contains fat, Grade 2: >50% of cross-section (left plus right side) contains fat

**Table 4:** Number of levels registered with fat (grade 1 or 2) in the multifidus muscles at pre-treatment in the two analysis- / treatment groups

	Predictor analysis (*n* = 147)		Analysis of change in fat infiltration (*n* = 126)	
	Rehab (*n* = 64)	Surgery (*n* = 83)	Rehab (*n* = 63)	Surgery (*n* = 63)
0 levels with fat (n / %)	31 (48.4)	36 (43.4)	30 (47.6)	29 (46.0)
1 level with fat (Grade^a^ 1 or 2; n / %)	18 (28.1)	24 (28.9)	18 (28.6)	19 (30.2)
2 levels with fat (Grade^a^ 1 or 2; n / %)	12 (18.8)	18 (21.7)	13 (20.6)	11 (17.5)
3 levels with fat (Grade^a^ 1 or 2; n / %)	3 (4.7)	5 (6.0)	2 (3.2)	4 (6.3)

^a^Grading according to the criteria by Kjaer et al [9] and Solgaard et al [31]: Grade 0: 0 or < 20% of total cross-section (left plus right side) contains fat, Grade 1: 20%–50% of cross-section (left plus right side) contains fat, Grade 2: >50% of cross-section (left plus right side) contains fat

In explorative comparison of baseline and 2-year clinical outcome (pain, ODI, work status) between patients with grade 0 *versus* grade 1–2 pre-treatment multifidus muscle fat in the two treatment groups, patients in the surgical group with fat grade 0 had better 2 year values for ODI and work status (but not pain). No such findings were seen in the rehabilitation group (Table 5). Unadjusted regression analysis revealed that patients with grade 0 pre-treatment multifidus muscle fat in the surgery group had significantly better ODI and work status after 2 years. Further strengthening this finding, grade 0 pre-treatment muscle fat was significantly related to lower pain scores in the surgery group at 2-year follow-up after adjusting for age, gender, BMI, smoking, and leisure time physical activity (and baseline pain/ODI in analysis of 2-year pain/ODI as dependent variable). The regression analysis showed no significant results for patients treated with rehabilitation (Tables 6 and 7). Analysis of normality, homoscedasticity, collinearity, and VIFs did not reveal any violation of these factors to the assumptions of the models.

**Table 5:** Exploring pain, ODI, and work status in patients with grade 0 *versus* grade 1–2 multifidus muscle fat in patients included in the predictor analysis^a^

	Rehabilitation			Surgery		
	Grade 0 fat at pre-treatment (*n* = 31)	Fat grad 1-2 at pre-treatment (*n* = 33)	*p*-value	Grade 0 fat at pre-treatment (*n* = 36)	Fat grade 1-2 at pre-treatment (*n* = 47)	*p*-value
Pain baseline (mean (SD))	75.1 (11.7)	70.8 (14.2)	0.19*	70.5 (15.6)	65.5 (15.8)	0.16*
Pain 2 year (mean (SD))	50.4 (28.9)	42.8 (26.5)	0.29*	25.9 (28.2)	33.3 (27.1)	0.24*
ODI baseline (mean (SD))	42.8 (8.6)	41.8 (8.0)	0.65*	40.0 (8.0)	44.1 (10.6)	0.05*
ODI 2 year (mean (SD))	28.9 (15.1)	25.5 (12.3)	0.33*	15.0 (17.1)	22.4 (14.6)	0.04*
Work status baseline (n / % working)	4 (12.9)	7 (21.2)	0.51#	12 (33.3)	8 (17.4)	0.12#
Work status 2 year (n / % working)	15 (48.4)	11 (33.3)	0.11#	24 (72.7)	22 (46.8)	0.04#

*ODI* Oswestry Disability Index

*Independent-samples t-test

#Chi-Square Test (Continuity Correction)

^a^ *n* = 144 for pain, *n* = 147 for ODI, *n* = 137 for work status

**Table 6:** Multiple regression analysis (unadjusted and adjusted) of effect of grade 1–2 pre-treatment multifidus muscle fat on pain and ODI at 2 years in each treatment group

		Pain			ODI		
		B	95% CI for β	*p*-value	B	95% CI for β	*p*-value
Rehab (*n* = 63 (pain)/64 (ODI))	Unadjusted	-7.56	-21.56–6.45	0.29	-3.36	-10.23–3.51	0.33
	Adjusted^a^	-5.93	-25.18–13.31	0.54	-1.49	-10.46–7.48	0.74
Surgery (*n* = 81 (pain)/83 (ODI))	Unadjusted	7.40	-4.93–19.73	0.24	7.35	0.40–14.29	0.04
	Adjusted^a^	15.36	0.92–29.79	0.04	10.39	2.50–18.28	0.01

*ODI* Oswestry Disability Index

^a^The model is adjusted for age, gender, body mass index, smoking, and leisure time physical activity. In addition, the model for 2-year pain is adjusted for baseline pain, and the model for 2-year ODI is adjusted for baseline ODI

**Table 7:** Logistic regression model (unadjusted and adjusted) predicting likelihood of working at 2 years in each treatment group

		*p*-value	B	OR	95% CI for OR	
					Lower	Upper
Rehab (*n* = 58)	Unadjusted	0.08	0.957	2.603	0.896	7.563
	Adjusted^a^	0.25	0.779	2.179	0.575	8.261
Surgery (*n* = 79)	Unadjusted	0.03	1.068	2.909	1.114	7.598
	Adjusted^a^	0.03	1.357	3.886	1.107	13.638

*OR* odds ratio, *CI* confidence interval

^a^The model is adjusted for age, gender, body mass index, smoking, and leisure time physical activity

More patients had increased multifidus muscle fat in the surgical group at 2-year follow-up than in the rehabilitation group (11.1%, 7 of 63 patients vs. 1.6%, 1 of 63 patients, *p* = 0.03, Mid-P exact test, 2x2 table for increase *versus* reduction or no change; raw data shown in Table 8). The difference remained but was not significant (*p* = 0.08) in the per protocol analysis. Explorative analysis revealed that clinical outcomes in the surgery group at 2-year follow-up were worse for patients with increased multifidus muscle fat *versus* those without (Table 9). The differences remained significant for pain (*p* < 0.01) and ODI (*p* = 0.03) in the per protocol analysis. Another explorative analysis showed that the difference in pain and ODI was present already 6 weeks postoperatively (data not shown, *p* = 0.06 (pain) and *p* < 0.01 (ODI), independent-sample t-test).

**Table 8:** Change in multifidus muscle fat in the two treatment groups from pre-treatment to 2-year follow-up

	Rehabilitation (*n* = 63)	Surgery (*n* = 63)
Improvement in 1 level	1^a^	0
No change	61	56
Deterioration in 1 level	1^b^	5^c^
Deterioration in 2 levels	0	2^d^

^a^Change from grad 1 to grade 0

^b^Change from grade 0 to grade 1

^c^All changes were from grade 0 to grade 1

^d^One patient changed from grade 0 to grade 1 in two levels, one patient changed from grade 0 to grade 1 in one level and from grade 1 to grade 2 in one level

**Table 9:** Clinical outcome at 2-year follow-up in the surgery group for patients with increased multifidus muscle fat *versus* those without

	No change in multifidus muscle fat	Increased multifidus fat in 1 or 2 levels	*p*-value
Pain at 2 year (mean (SD)) (*n* = 54/7)	29.2 (26.2)	63.0 (33.5)	<0.01*
ODI at 2 year (mean (SD)) (*n* = 56/7)	16.8 (14.2)	42.6 (20.3)	<0.001*
Work status at 2 year (n/ % working) (*n* = 55/6)	36 (65.5)	1 (16.7)	0.03#

*ODI*, Oswestry Disability Index

*Independent-sample t-test

#Mid-P exact test

## Discussion

This study on multifidus muscle fat had three main findings. First, less fat on pre-treatment MRI predicted better 2-year clinical outcomes after TDR surgery (i.e. more fat predicted worse outcomes). Second, more patients had increased fat at 2-year follow-up in the surgery group than in the rehabilitation group. Third, increased fat at 2-year follow-up was related to a less favorable clinical outcome in the surgical group.

### Discussion of findings

Less pre-treatment multifidus muscle fat was also related to a better clinical result (lower ODI, i.e. better function) at 2-year follow-up after TDR surgery in the only former study on this issue [18]. This indicates that less multifidus muscle fat is favorable prior to TDR surgery. Exercise can prevent fat infiltration of other muscles [17] and might perhaps help to prevent multifidus muscle fat as well. Exercise science states that muscular strength reduces the risk of developing functional limitations [39]. A recent report lists no and low physical activity as risk factors for disability [40]. Further, low physical activity is found to be associated with fat in the multifidus muscles in a dose-dependent manner [15]. Presence of pre-treatment fat may indicate physical inactivity not caught by our categorical leisure time physical activity variable controlled for in the analysis. Less favorable clinical outcome in patients with grade 1 or 2 pre-treatment fat in the surgical group might also be caused by pain-induced alterations of paraspinal morphology not solved by surgery. It is hypothesized that pain-induced muscular alterations is caused by long-loop inhibition of the multifidus together with a combination of reflex inhibition and substitution patterns of the trunk muscles [10]. Localized multifidus morphology changes corresponding to painful levels has been described previously [10–12]. Similar hypotheses are postulated for other muscle groups [41–43].

Lack of structured post-operative rehabilitation and possible post-operative inactivity may partly explain why increased multifidus muscle fat at 2-year follow up was more common in the surgery group than in the rehabilitation group. The surgery group did not receive post-operative rehabilitation by routine and may have tended to remain inactive, whereas the rehabilitation group received comprehensive general and specific functional and muscular restoration and was encouraged to continue exercising and being active after the rehabilitation program ended [3, 32]. This may also explain maintenance of muscle morphology in all but one patient in the rehabilitation group. Exercises have proved useful for maintaining and improving muscle condition in patients with LBP [11, 20, 44–46]. Additionally, the surgery itself may have induced muscular alterations. Biomarkers have indicated general muscle atrophy following surgery [47] and atrophy of back muscles has been reported after lumbar interbody fusion surgery [22, 24, 25]. Another explanation may be neuromuscular deficits as reported following other surgical techniques that cause minimal muscle damage [41, 42]. Our finding of increased multifidus muscle fat in the surgery group at 2-year follow-up should be assessed in further studies, also because all increases in fat infiltration were of only one grade (from grade 0 to grade 1, or grade 1 to grade 2; footnote Table 8). The finding was weakened in the per protocol analysis, perhaps due to few cases (*n* = 7) with increased fat.

Our explorative analyses indicated that increased multifidus muscle fat in the surgical group at follow-up was related to a worse clinical outcome. Interestingly, the difference in pain and ODI reported at 2-year follow-up between patients with and without increased fat in the surgery group is considerable (33.8 mm for pain and 25.8 points for ODI respectively, Table 9) and well above suggested limits for clinically important outcomes for differences in pain (20 mm) and ODI (10 points) [48]. The worse pain being present already 6 weeks postoperatively may have contributed to increased muscular alterations [10, 12, 25, 41–43]. Again, since only 7 patients in the surgery group had increased fat, these results should be interpreted with caution and ought to be re-examined in further studies. Still it is possible that severe pain and reduced mobility among these few patients led to increased fat infiltration.

### Strengths and limitations

MRI is a valid method for evaluating muscle fat infiltration [49]. In our study, three experienced radiologists blinded to clinical data performed independent evaluations so that none of them had undue influence on the results [50]. The interobserver agreement was only moderate but the use of multiple readers likely increased the consistency of the conclusive ratings compared to studies with a single reader [51]. The direct comparison of post- and pre-treatment images, as in routine clinical practice, is the preferred method for evaluating changes in MRI findings over time [52–54]. It may reduce erroneous rating of changes due to ambiguous findings or minor differences in MRI technique, and can provide a more reliable rating (moderate to very good interobserver agreement) than separate evaluations of post- and pre-treatment images [52]. We used MRI rather than computed tomography, since MRI is without radiation exposure and can provide better soft tissue resolution and contrast and slightly more reliable muscle evaluations [55]. Muscle fat was graded visually also in former studies [9, 18]. However, single-voxel proton MR spectroscopy detects smaller fat amounts not visible on conventional MRI; this method identified more fat in the multifidus muscles in chronic LBP patients than in asymptomatic volunteers, despite no difference was seen on conventional MRI [49]. Hence, results might have differed had we used alternative or more sophisticated fat evaluation methods.

The study had a well-defined sample of patients with chronic non-specific LBP and localized MRI findings, and it included the three most important outcome variables for evaluating LBP patients [56, 57]. The follow-up rate was fair: 79–85% (137-147/173) had 2-year data for the predictor analysis and 73% (126/173) had data for comparing change in fat infiltration over time between treatment groups. Our study design allowed, for the first time within the field, comparisons of MRI findings between patients treated with and without surgery. According to the literature, the length of follow-up is sufficient to evaluate change in muscle morphology over time [16]. Our regression models included only one candidate predictor, five other variables, and ≥137 patients (the adjusted models lacked data on some variables). The models were therefore well within the recommended limit of at least ten observations for each exposure variable studied [58]. The decision to analyze patients according to as-treated-principles in the predictor analysis was based on our *a priori* decided research questions. We could have analyzed patients in a single merged cohort and controlled for treatment group in the regression models, but this procedure may be more relevant with multiple clinical questions. We could also have controlled for other potential effect moderators, as we know that diabetes mellitus and cardiovascular disease can affect muscle fat [59, 60], but only 20% of patients had comorbidities. We could not compare muscle fat to muscle function, which was not tested. The significance level of 0.05 in multiple explorative analyses implied a risk of wrong conclusions. Finally, smaller differences and changes in muscle fat (and perhaps more convincing associations) might have been detected if more categories (or a continuous measure) of fat had been used and/or MRI to assess fat had been performed more than once during the follow-up period.

### Potential implications

Better outcome for patients with less multifidus muscle fat before treatment may be a result of a better starting point and a clinical implication could be that patients scheduled for TDR surgery should optimize their back muscle condition before surgery. Since we found worse outcome in patients with increased muscle fat at 2-year follow-up after TDR surgery, postoperative rehabilitation may also be relevant. This may be supported by a study of patients receiving back fusion [61] and may be especially relevant for those with substantial postoperative back pain. However, our findings should be re-examined in further studies.

## Conclusions

In this secondary analysis of data from a randomized trial comparing clinical efficacy of multidisciplinary rehabilitation versus TDR surgery, patients with less fat infiltration of multifidus muscles before TDR surgery had better outcomes at 2-year follow-up. Our findings also indicated a negative influence of TDR surgery on back muscle morphology in some patients. The rehabilitation group maintained their muscular morphology and were unaffected by pre-treatment multifidus muscle fat.

## Abbreviations

BMI: Body mass index
DICOM: Digital Imaging and Communications in Medicine
HIZ: High intensity zone
ITT: Intention-to-treat
LBP: Low back pain
MRI: Magnetic resonance imaging
ODI: Oswestry Disability Index
TDR: Total disc replacement
VAS: Visual analogue scale
